# Multi-Response Optimization of Abrasive Waterjet Machining of Ti6Al4V Using Integrated Approach of Utilized Heat Transfer Search Algorithm and RSM

**DOI:** 10.3390/ma14247746

**Published:** 2021-12-15

**Authors:** Kishan Fuse, Rakesh Chaudhari, Jay Vora, Vivek K. Patel, Luis Norberto Lopez de Lacalle

**Affiliations:** 1 Department of Mechanical Engineering, School of Technology, Pandit Deendayal Energy University, Raysan, Gandhinagar 382007, India; kishan.fuse@sot.pdpu.ac.in (K.F.); jay.vora@sot.pdpu.ac.in (J.V.); vivekp@sot.pdpu.ac.in (V.K.P.); 2 Department of Mechanical Engineering, University of the Basque Country, Escuela Superior de Ingenieros Alameda de Urquijo s/n., 48013 Bilbao, Spain

**Keywords:** abrasive waterjet machining (AWJM), Ti6Al4V, response surface methodology (RSM), optimization, heat transfer search (HTS) algorithm, surface morphology

## Abstract

Machining of Titanium alloys (Ti6Al4V) becomes more vital due to its essential role in biomedical, aerospace, and many other industries owing to the enhanced engineering properties. In the current study, a Box–Behnken design of the response surface methodology (RSM) was used to investigate the performance of the abrasive water jet machining (AWJM) of Ti6Al4V. For process parameter optimization, a systematic strategy combining RSM and a heat-transfer search (HTS) algorithm was investigated. The nozzle traverse speed (T_v_), abrasive mass flow rate (A_f_), and stand-off distance (S_d_) were selected as AWJM variables, whereas the material removal rate (MRR), surface roughness (SR), and kerf taper angle (θ) were considered as output responses. Statistical models were developed for the response, and Analysis of variance (ANOVA) was executed for determining the robustness of responses. The single objective optimization result yielded a maximum MRR of 0.2304 g/min (at T_v_ of 250 mm/min, A_f_ of 500 g/min, and S_d_ of 1.5 mm), a minimum SR of 2.99 µm, and a minimum θ of 1.72 (both responses at T_v_ of 150 mm/min, A_f_ of 500 g/min, and S_d_ of 1.5 mm). A multi-objective HTS algorithm was implemented, and Pareto optimal points were produced. 3D and 2D plots were plotted using Pareto optimal points, which highlighted the non-dominant feasible solutions. The effectiveness of the suggested model was proved in predicting and optimizing the AWJM variables. The surface morphology of the machined surfaces was investigated using the scanning electron microscope. The confirmation test was performed using optimized cutting parameters to validate the results.

## 1. Introduction

Owing to excellent advantageous properties such as a low elastic modulus, high corrosion resistance, excellent strength, weldability, and heat-treatable nature, the titanium Ti6Al4V alloys have become more popular in widespread application areas such as automobiles, jet engines, power-generating components, body frame of aircraft, and medicated implants [1,2]. Due to the high fatigue strength and fracture-resistant characteristics of these metals, they exhibit wide applications [3,4]. The engaging explicit qualities of Ti6Al4V alloys broaden its use in various fields [1]. However, the poor thermal conductivity of titanium alloys challenges its machinability in conventional manufacturing techniques [5,6,7]. The poor conduction of heat causes attainment of higher temperatures during conventional machining of the alloy resulting in significant tool wear and hence higher machining cost [8,9,10]. Keeping in mind the tool wear of hard-to-cut titanium alloy, past researchers have explored various non-conventional techniques to overcome the difficulties associated with traditional machining [6,11,12,13,14,15].

Abrasive waterjet machining (AWJM) is one of the most widely used non-traditional processes in industries for the machining of hard materials [14,16,17]. AWJM has a high cutting speed, ensures high accuracy and flexibility, has no heat-affected zone, and is eco-friendly [18,19]. Some of the other advantages include the low machining cost, ease in programming, and conservation of properties due to a lower temperature during machining and the wide range of machinable materials [15,19,20]. In AWJM, the high velocity and pressured water jet mixed with abrasives target the workpiece leading to erosion of the material. Some of the limitations of AWJM include the development of surface roughness, kerf taper, delamination, abrasive embedment, etc., which results in poor quality of the machined part [18]. The use of optimized AWJM process parameters can reduce these limitations.

Several researchers have studied the optimization of the AWJM process parameters. Chaturvedi et al. [6] investigated the optimization of AWJM variables for machining of Ti6Al4V alloy. They performed experiments using an L_25_ orthogonal array considering the pressure, stopping distance, transverse speed, and abrasive throughput as process variables and surface roughness, MRR, machining time, HRC strength of the machined part as a response. They found pressure to be the most significant parameter affecting machining time and surface roughness. Saravanan et al. [1] employed the Taguchi-gray relational technique for optimizing AWJ machining parameters for Ti6Al4V alloy. They considered SiC volume, SiC size, and abrasive flow rate (A_f_) as potential parameters affecting MRR and SR and found that particle size was the significant variable for producing higher MRR with low SR. Karkalos et al. [14] studied AWJ machining on Ti6Al4V alloy using glass beads as abrasives to investigate the economic and environmental score of sustainability for the process. To determine the correlation between input variables and the depth of penetration, kerf taper angle, and kerf width, they performed GRA. They reported AWJM as a sustainable process. Tripathi et al. [21] investigated the effect of cutting speed and A_f_ on MRR, SR, roundness, and cylindricity. Their optimized results observed that a recently developed Rao algorithm was more effective compared to JAYA and TLBO algorithms. Patel et al. [22] explored AWJM for the machining of polymer matrix composites using four multi-criteria techniques, namely MOORA, TOPSIS, GRA, and Data Envelopment Analysis-based Ranking (DEAR). They found that the optimal setting determined using the DEAR method resulted in better quality characteristics.

Thakur and Singh [18] investigated the optimization of SR, MRR, and the delamination factor during AWJM by considering the MWCNT weight percentage, stand-off distance (S_d_), jet pressure, and nozzle traverse rate (T_v_) as input variables. The optimized settings using GRA resulted in 4.25% improved MRR, a reduction of 23.94% in kerf taper, and a reduction of 26.08% in the delamination factor compared to the initial set of parameters. The machining of aluminum/tungsten carbide composites using AWJM was optimized by Kumar et al. [23]. They prepared specimens using 2, 4, 6, 8, and 10 wt% of tungsten carbide. RSM was implemented for developing a mathematical relationship between dependent and independent variables. ANOVA results highlighted transverse speed as the most influential variable on MRR followed by % reinforcement and S_d_. Joel et al. [24] used Multi-Objective Teaching Learning-Based Optimization (MOTLBO) to optimize three conflicting responses as the minimization of SR and the maximization of MRR and hardness during machining of C360 brass using AWJM. Deaconescu and Deaconescu [9] deployed the Box–Behnken design of RSM during cutting of X2 CrNiMo 17-12-2 austenitic stainless steel using AWJM. They obtained good surface quality using a lower traverse speed and S_d_, and medium to fine grit size.

Doğankaya et al. [25] presented the parametric effect of AWJM variables on UHMWPE plates. For experimental design, they used CCD of RSM. ANOVA results highlighted effective parameters among the abrasive mass flow rate, pressure, S_d,_ and feed. They applied particle swarm optimization to find a trade-off between conflicting response measures such as surface roughness and dimensional error. The reported accuracy of regression models varied between 0.1% and 5.6%. Reddy et al. [26] investigated multi-objective optimization using WASPAS and MOORA techniques for input controllable parameters of AWJM such as T_v_, A_f_, and S_d_, which influences performance characteristics of MRR, kerf width, and SR. They performed experiments on the Inconel-625 workpiece. They found that MRR was positively varying with T_v_ and A_f_. SR was increasing with an increase in A_f_ and decreasing with an increase in T_v_. Samson et al. [27] identified optimum parameter settings in AWJM of Inconel 718 alloy using the VIKOR method. They identified a pressure of 180 MPa, A_f_ 0.42 kg/min, and S_d_ 2 mm as an optimum condition. The L9 orthogonal array was used to design experiments. The selected input variables were A_f_, S_d_, and pressure whereas the performance characteristics were MRR, SR, taper angle, and roundness.

The literature review revealed that parametric optimization of AWJM has been investigated to a great extent using several multi-objective techniques such as GRA, TOPSIS, VIKOR, WASPAS, MOORA, MOTLBO, JAYA, TLBO, etc. One of the revolutionary multi-objective algorithms is the Heat transfer search (HTS) algorithm [28]. The HTS algorithm is a parameterless optimization algorithm that is faster, easy to implement, and demonstrates better convergence towards the results. The major advantageous side of the HTS algorithm is the right balance of exploration and exploitation. Six different search mechanisms introduced in this algorithm make it properly balanced. The number of generations helps in generating the different search mechanisms. HTS results in a globally optimal solution with ease in solving critical problems. In recent times, the HTS has successfully been used for various benchmarking objectives pertaining to a variety of fields [9,29,30,31,32]. Patel and Raja [33] carried out a performance assessment of a heat pump using the HTS algorithm. The results of the algorithm were elucidated in the form of Pareto-optimal points. They compared optimized results with experimental results and found a deviation of 10.95% in the co-efficient of performance. Raja et al. [34] proposed the HTS algorithm for thermo-economic and thermodynamic optimization of the heat exchanger considering six design variables. They further compared the results of the HTS algorithm with NSGA-II and TLBO and found the HTS algorithm was more effective. Chaudhari et al. [35] optimized WEDM machining variables using an RSM-based HTS algorithm. They used the BBD of RSM for the experimental design with microhardness, MRR, and SR as output attributes. The generated models were verified using ANOVA. They presented a set of optimal non-dominant solutions. Vora et al. [29] optimized the process parameters of the activated-tungsten inert gas welding process using the HTS algorithm. The obtained results from the study have shown good agreement between the predicted and actual output of responses. Vora et al. [9] adopted the HTS algorithm to generate optimal Pareto points during laser cutting of Ti6Al4V alloy. The designing of experiments was accompanied using Taguchi’s L_9_ array. They generated 2D and 3D Pareto graphs for selecting optimal input variable settings for multiple responses.

However, to the best of the author’s knowledge, no study has been reported on the application of HTS for optimizing performance characteristics of the AWJM process for Ti6Al4V alloy. Thus, the present study has conducted an exhaustive investigation to simultaneously optimize MRR, SR, and the Kerf taper angle using the HTS algorithm. RSM with the analysis of variance is used to develop the significant mathematical relationship between input and output variables. The significance of the variables was tested at a 95% confidence level using the ANOVA technique, which is essential to recognize the most influential model terms. The effect of machining variables on responses was then studied by plotting the main effect plots for individual response variables. Single-objective and multi-objective optimization was then carried out using the HTS algorithm. Confirmation trials have been conducted to check the accuracy of predicted results of the HTS algorithm. The authors strongly believe that the present study will provide significant information to the researchers and industries working with AWJM.

## 2. Materials and Methods

**Figure 1:**
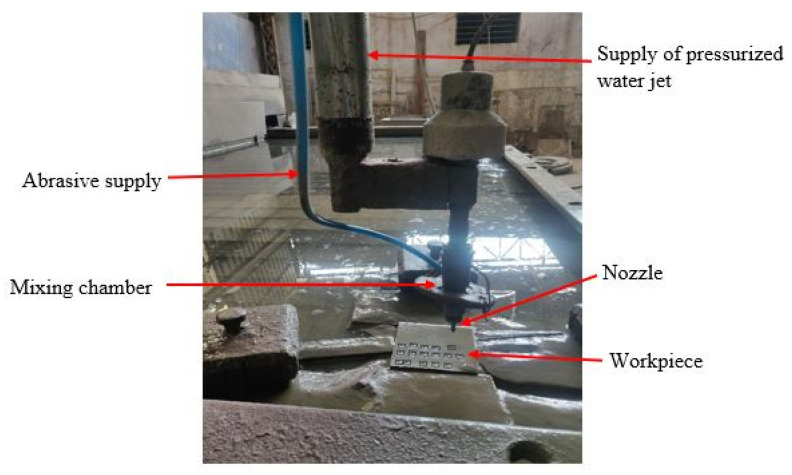
Experiential setup of AWJM process used in current study.

The experimental work was carried out using a 3-axis jetcut 1631 abrasive waterjet cutting machine (Innovative International Ltd., Ahmedabad, India). The workpiece Ti6Al4V alloy was fixed on the machine bed using fixtures. The workpiece was procured from Nextgen steel and alloys, Mumbai, India. The chemical composition of the workpiece examined by spectroscopic analysis is given in Table 1. The machine was fitted with a 0.1 mm diameter nozzle and controlled using a fanuc controller. The abrasive material used during experiments was silicon carbide. Figure 1 demonstrates the working configuration during experiments. The selected input process factors were nozzle traverse speed (T_v_), abrasive flow rate (A_f_), and stand-off distance (S_d_). The levels of variable process parameters, as shown in Table 2, were assigned based on published literature for similar alloys and a series of recurrent tests. Table 2 also shows the machining conditions. MRR, SR, and the kerf taper angle (θ) were considered as the quality characteristics.

The experiments were systematically designed using the design of experiments (DOE) technique. The DOE arranges the experiments such that the maximum amount of information can be collected by performing fewer experiments. There are many experimental designs are available under the DOE heading. Among them, the most popular are Box–Behnken designs based on RSM [30]. RSM is a mathematical modeling technique used to build a relationship between dependent and independent variables [35]. The BBD for three process parameters, each at three levels, was designed for the presented study using Minitab 17 statistical analysis software. A total of 15 experiments were designed using BBD. Square slots 10 mm in size were created in a Ti6Al4V alloy with small uncut material at the exit such that the slot remains intact with the base plate. Table 3 shows the 15 experimental runs by considering the 3 factors at 3 levels.

The MRR (gram/s) is the quantum of material removed per unit of time and is calculated using Equation (1). The weight of the job before machining (W_bm_) and after machining (W_am_) was determined using a digital weighing machine with a precision of ±0.001 g. The time (t) required for cutting the square slot of the defined size is measured using a stopwatch with an approximate precision of ±1 s.

(1) $$\mathrm{MRR}=\frac{W_{\mathrm{bm}}-{\;W}_{\mathrm{am}}\;}{\rho \times t}$$

The surface roughness was measured with the Mitutoyo Surftest SJ-410 model (Mitutoyo Ltd., New Delhi, India) with a precision of ±0.01 µm. The cutoff length selected was 0.8 mm with an evaluation length of 8 mm. Three measurement runs were conducted, and the average value was considered for further calculation.

**Figure 2:**
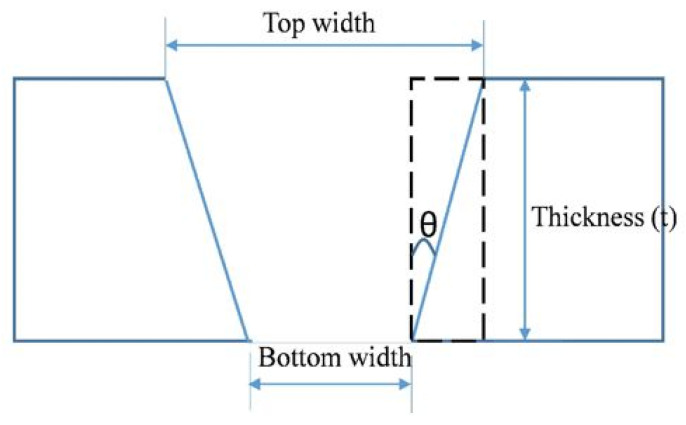
Representation of the Kerf taper angle.

The kerf taper angle was calculated using Equation (2).

(2) $$\theta ={\;\tan}^{-1}\left[\frac{W_{t}-W_{b}}{2d}\right]$$

where W_t_ is the width at the top, W_b_ is the width at the bottom, and d is the depth of penetration. The top width and bottom width of the machined slot were measured using optical microscopy. Figure 2 shows the schematic of the kerf taper angle measurement.

A scanning electron microscope (Zeiss Ultra 55, Bangalore, India) was used to investigate the surface morphology of the machined surfaces. The experimental data were then analyzed using Minitab software (version 17). The effect of process variables in terms of their contribution was investigated using ANOVA. A confidence interval of 95% (α = 0.05) was considered for analysis.

The selected-response MRR is of the ‘higher the better’ category whereas SR and the kerf taper angle are in the ‘lower the better’ category. So, it is essential to determine the optimal parametric settings for achieving these responses simultaneously. According to this, optimization of the AWJM variables was performed in the present study by the HTS algorithm for determining optimal parameter setting.

## 3. Results and Discussion

The AWJM process variables as per the BBD of RSM are shown in Table 3. The measured values of the selected output response parameters of MRR, SR, and θ are also shown in Table 3. Mathematical regression models were generated using the RSM technique for the prediction of output responses. ANOVA was used for statistical analysis, which shows the influence of machining variables on output parameters. Minitab 17 software was used for all the statistical analyses. The significance of the variables was tested at a 95% confidence level, which is essential to recognize the most influential model terms [30]. For a confidence level of 95%, the *p*-value for any input parameter should be less than 0.05 to consider the respective parameter term as significant [36,37]. The effect of the machining variables on responses was then studied by plotting the main effect plots for individual response variables. The main effect plots of the response highlight the optimum factor-level combinations for a given response. Single-objective and multi-objective optimization was then attempted by the HTS algorithm followed by the confirmation experiments.

### 3.1. Analysis of MRR

The relative effect of machining variables on responses can be effectively determined using the ANOVA technique. The investigation of input process parameters on MRR was determined by *p*-value at a 95% confidence level. The *p*-value for any input parameter should be less than 0.05 to consider the respective parameter term as significant at the 95% level [38]. A non-linear regression equation was developed using the backward elimination method, with α selected as 0.05 for a 95% significant level. So, the non-significant terms were eliminated from the regression equation as they did not have any significant impact on the response value. Equation (3) shows the mathematical regression equation for MRR developed using Minitab 17 software through the backward elimination procedure.

(3) $$\mathrm{MRR}\;=-0.2395+0.000589\;\left(T_{v}\right)+0.00069\;\left(A_{f}\right)+0.0861\;\left(S_{d}\right)-0.000196\;\left(T_{v}\;\times \;S_{d}\right)-0.000104\;\left(A_{f}\times S_{d}\right)$$

where T_v_ is the nozzle transverse speed, A_f_ is the abrasive flow rate, and S_d_ is the stand-off distance.

**Figure 3:**
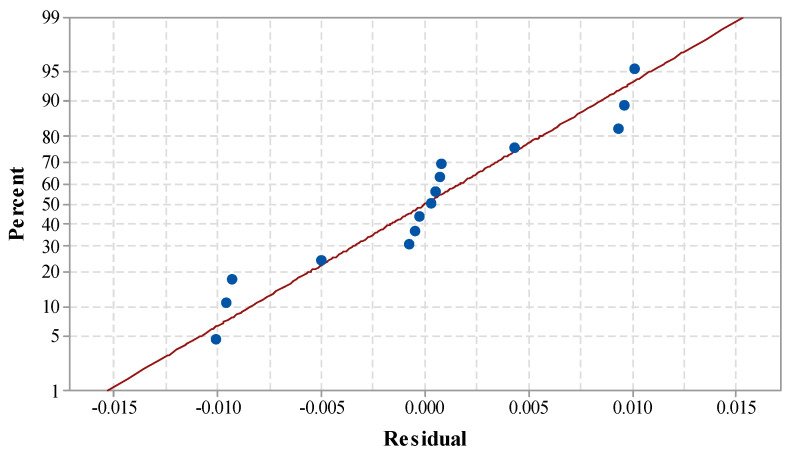
Normal probability plot for MRR.

Table 4 shows the ANOVA for MRR. The *p*-value of the model term was obtained as 0.000, which is less than 0.05. This suggests the model has a large significant effect. The linear model term along with all three input process parameters was found to have a significant effect on MRR. Lack-of-fit was obtained as an insignificant term, which indicates that the proposed model is adequate for predicting the output variables [28,35]. Insignificant lack-of-fit also reveals the adequacy and fitness of the proposed model [35]. The value of R² indicates that 96.27% of the variation of MRR was contributed by the control factors, and only 3.63% of the total variation cannot be described by the proposed model. The ‘Adj. R-Sq.’ was obtained as 94.19%. The proposed model can be treated as adequate and the best fit, as the variation between R-sq. and Adj. R-sq. was obtained to be within the limit [39,40,41]. The close relation of the R-square values signifies the model is appropriate for predicting the future outcomes of MRR [42]. The standard deviation of 0.008341 was obtained for MRR. This shows the maximum deviation can only be 0.008341 from the mean value. Figure 3 shows the normal probability plot for MRR. It can be observed that all the residuals are positioned on a straight line. This indicates the suitability of the existing model for the future outcome, and the regression model is also well fitted with the observed values [43].

**Figure 4:**
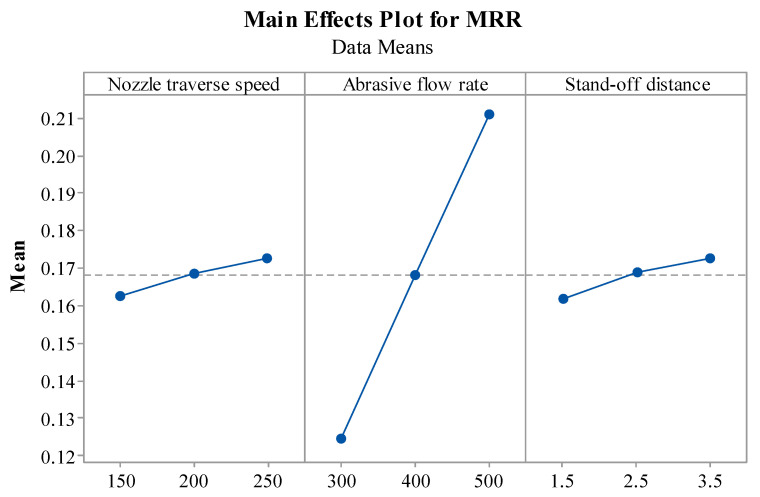
Effect of AWJM parameters on MRR.

Figure 4 represents the influence of various parameters on MRR. It can be observed that T_v_ increases the MRR. This is due to an increase in intermolecular forces and energy causing the sharing and erosion of more material from the parent material [23]. Higher T_v_ allows very little overlap of the machining action thus increasing MRR [44]. It can be highlighted that when A_f_ is increased from 300 to 500 gm/min, it enhanced the rate of material removal. The abrasives have sharp edges, which perform the cutting action. As the number of abrasives increase with an increase in A_f_, the number of sharp edges performing the cutting action increases, resulting in higher MRR. Furthermore, higher A_f_ allows more abrasive particles to penetrate the surface, thus increasing MRR [45]. An increase in S_d_ also enables higher MRR due to the divergence of the jet because the jet diameter increases due to divergence, which leads to the erosion of material from larger areas [18]. Reddy et al. [45] reported that MRR increases with three process variables, namely, T_v_, A_f_, and S_d_ which coincide with the results obtained in the present study.

### 3.2. Analysis of SR

Statistical analysis using ANOVA to investigate the effect of input process parameters on SR is shown in Table 5. The mathematical regression equation for SR is developed using the backward elimination procedure as shown in Equation (4):

(4) $$\mathrm{SR}\;=9.08-0.01764\;\left(T_{v}\right)-0.01072\;\left(A_{f}\right)+1.947\;\left(S_{d}\right)+0.000087\;\left(T_{v}\;*\;T_{v}\right)+0.1928\;\left(S_{d}\times S_{d}\right)+0.003269\;\left(A_{f}\times S_{d}\right)$$

**Figure 5:**
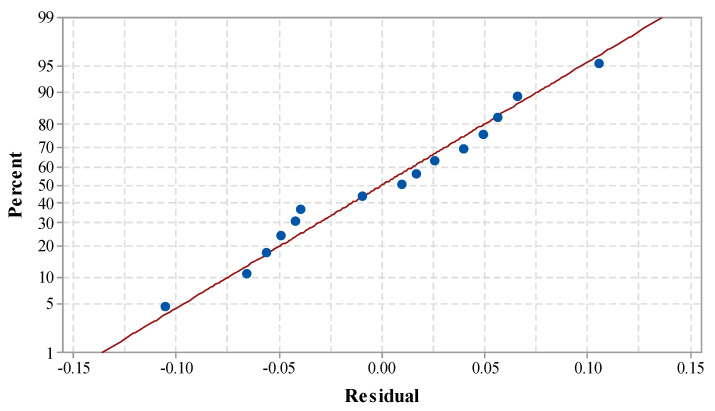
Normal probability plot for SR.

The ANOVA results for the developed model at a 95% confidence level are shown in Table 5. The *p*-value of the model term of 0.000 suggests the existing model for SR is largely significant. The linear model, square model, and two-way interaction terms had a significant effect on SR. The process parameters of A_f_, S_d_ along with the interaction terms of T_v_ × T_v_, S_d_ × S_d_, and A_f_ × S_d_ had a significant effect on the response value. The ANOVA results of SR depict that SR is highly influenced by variation in A_f_ followed by S_d_. Lack-of-fit was obtained as an insignificant term, which indicates the proposed model is adequate for predicting the output variables [30]. Insignificant lack-of-fit also reveals the adequacy and fitness of the existing proposed model. The values of R-sq. and Adj. R-sq. were found to be 99.01% and 98.27%, respectively. An extremely close relation between these R-sq. values show the adequacy and fitness of the existing model. A standard deviation of 0.0991 shows the existing model is well suited for the prediction of future outcomes with the least error. Figure 5 shows the normal probability plot of SR. It can be observed that all the residuals are positioned on a straight line. The absence of residual clustering and a normal distribution of errors indicates the existing model is very well suited for predicting the satisfactory effect of the response [43].

**Figure 6:**
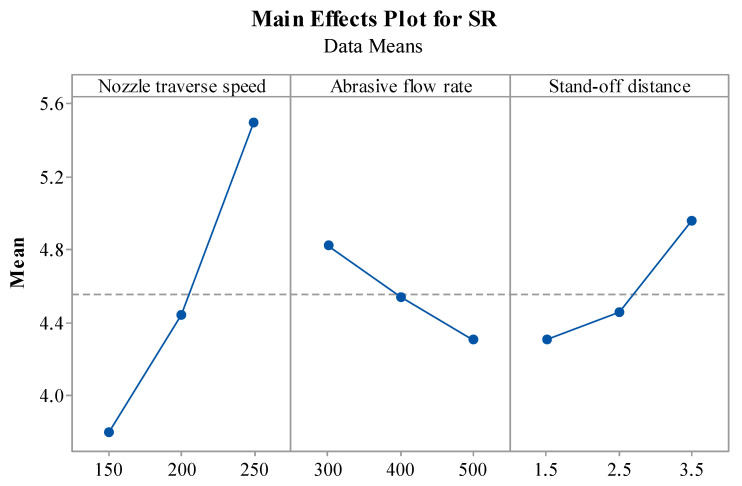
Effect of AWJM parameters on SR.

The influence of process parameters on SR is shown in Figure 6. Figure 6 shows an increase in T_v_ speed increases the SR. The reason for this is that at lower T_v_, a large number of abrasive particles are allowed more time to participate in the cutting action and this helps in removing a large number of asperities from the surface, resulting in a better-quality machined surface [20]. The trend of T_v_ and SR was in agreement with the trend reported by Deaconescu and Deaconescu while machining using AWJM [18]. In the case of A_f_, a decrease in SR can be observed with the increase in A_f_. In line with this, the smoothing of machined surfaces can result when A_f_ is increased [46]. In the case of S_d_, a remarkable increase in SR is seen when S_d_ increased from 1.5 to 3.5 mm. The divergence of the impacting jet before impingement caused by an increased S_d_ can be attributed to the observed phenomenon. At lower S_d_, coherence of the abrasive jet is maintained, which ensures high kinetic energy at the jet impact region, thus removing the asperities from the surfaces properly. However, at high S_d_, the jet becomes divergent resulting in a low density of abrasive particles due to the expansion of the jet, which generates more random peaks and valleys on the surface due to singular particles, thus making it unable to remove the material smoothly from the machining zone and producing a rough surface [9,24]. Thus, it is desirable to have low S_d_, which maintains the kinetic energy of the jet and produces smoother surfaces.

### 3.3. Analysis of Kerf Taper Angle

Table 6 shows the ANOVA results of the kerf taper angle to examine the influence of AWJM variables. The mathematical regression equation for the Kerf taper angle is developed using the backward elimination procedure as shown in Equation (5):

(5) $$\theta =4.615-\;0.00906\;\left(T_{v}\right)-\;0.0045333\;\left(A_{f}\right)-\;1.058\;\left(S_{d}\right)\;+\;0.000045\;\left(T_{v}\;\times \;T_{v}\right)\;+\;0.1325\;\left(S_{d}\;\times \;S_{d}\right)\;+\;0.001348\;\left(A_{f}\;\times \;S_{d}\right)$$

**Figure 7:**
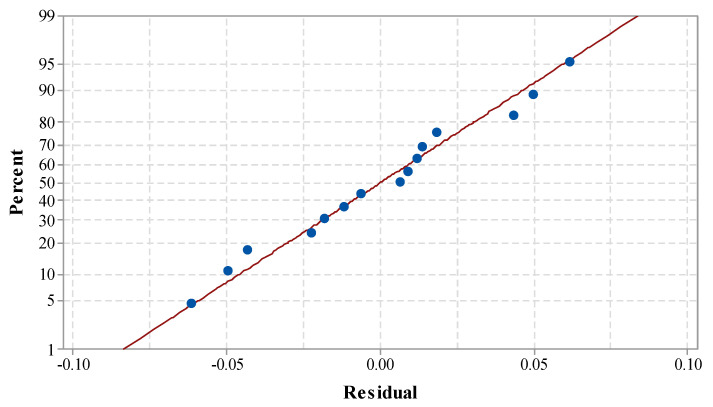
Normal probability plot for Kerf taper angle.

Table 6 shows the statistical results by ANOVA for the existing model of the kerf taper angle. The F-value of 111.22 along with a *p*-value of 0.000 of the model implies the developed model is significant. The linear model, square model, and two-way interaction terms were found to have a significant effect on the Kerf taper angle. The process parameters of A_f_, S_d_ along with the interaction terms of T_v_ × T_v_, S_d_ × S_d_, and A_f_ × S_d_ had a significant effect on the response value. ANOVA results of the kerf angle depict the kerf angle is highly influenced by variation in A_f_ followed by S_d_. The lack-of-fit value of 10.18 with a corresponding *p*-value of 0.092 implies an insignificant effect. An insignificant lack-of-fit suggests the adequacy and fitness of the proposed model [40]. The values of R-sq. and Adj. R-sq. were found to be 98.82% and 97.93%, respectively. An extremely close relation between these R-sq. values shows the adequacy and fitness of the existing model [38]. A standard deviation of 0.05 shows the existing model is well suited for the prediction of future outcomes with the least error. The normal probability plot of the Kerf taper angle is shown in Figure 7. It can be observed that all the residuals are positioned on a straight line. The absence of residual clustering and a normal distribution of errors indicates the existing model is very well suited to predicting the satisfactory effect of the response [9].

**Figure 8:**
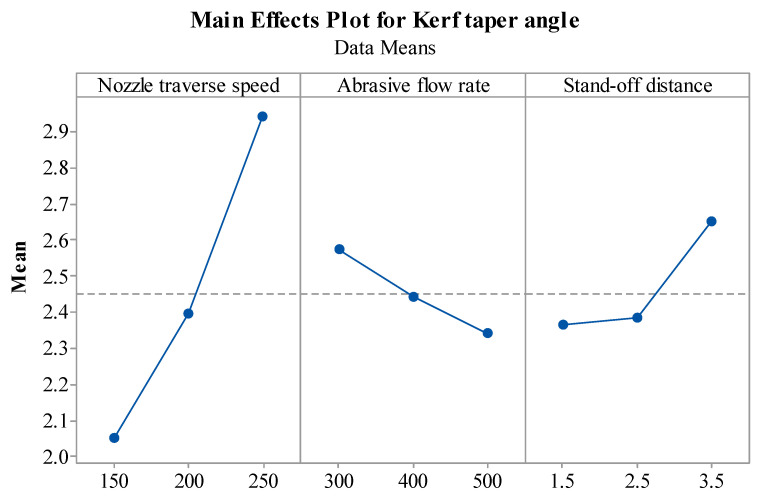
Effect of AWJM parameters for Kerf taper angle.

The kerf taper angle is a measure of the straightness of the machined slot cross-section. The main effect plot of the kerf taper angle as shown in Figure 8 indicated a positive correlation between T_v_ and the Kerf taper angle. Increasing the T_v_ increases the kerf taper angle. This can be attributed to insufficient broadening of the bottom kerf width by the jet as T_v_ increases. The A_f_ negatively affects the kerf angle. This can be explained by the fact that at higher A_f_, the kerf width value increases, which increases the kerf taper angle [10]. The increase in S_d_ was found to increase the taper angle. This can be attributed to the fact that at higher S_d_, the jet is impacted by the flaring mode. Thus, eroding more material at the top causes a higher top kerf width and lower bottom kerf width [46]. The results of the effect of A_f_ and S_d_ on the kerf taper angle obtained in the present study agree with the results reported by Dumbhare et al. [44].

The ANOVA analysis of all three responses showed that developed mathematical models are significant for predicting the responses. It indicates the experimental error is very minimal and collected output data can be used for multi-objective optimization.

### 3.4. Optimization Using HTS Algorithm

HTS is a population-based algorithm that mimics the thermal equilibrium behavior between the system and surroundings [47]. The algorithm is initiated by defining the population size, termination criteria, and upper and lower bounds of the design variable followed by the random solution of the initial population. The best solution of the random population is stored as the elite solution. Then the entire population undergoes any one of the heat-transfer phases (i.e., conduction, convection, and radiation) based on the probability parameter R to update the solution. The updated solution in the HTS algorithm is accepted only if it has a better functional value. Subsequently, the worst solutions of the population are replaced by the elite solutions. The updating mechanism of each of the phases is described in detail in the subsection below.

#### 3.4.1. Conduction Phase

Equations (6) and (7) are the equations that drive the update of solutions in the conduction phase [48],

(6) Xji′={Xk, i+(−R2Xk, i), iff(Xj)>f(Xk)Xj,i+(−R2Xj,i), iff(Xj)<f(Xk);ifg≤gmaxCDF 

(7) Xj,i′={Xk, i+(−riXk, i), iff(Xj)>f(Xk)Xj,i+(−riXj,i), iff(Xj)<f(Xk);ifg>gmaxCDF 

where $X_{j,i}^{\prime }$ is the updated solution; j = 1, 2,…, n; k is a randomly selected solution; j ≠ k; k ∈ (1, 2,…, n); i is a randomly selected design variable; i ∈ (1, 2,…, m); g_max_ is the maximum number of generations specified; CDF is the conduction factor; R is the probability variable; R ∈(0, 0.3333); r_i_ ∈ (0, 1) is a uniformly distributed random number.

#### 3.4.2. Convection Phase

The solutions are updated in the convection phase as per Equations (8) and (9) [48],

(8) $$X_{j,i}^{\prime }=X_{j,i}+R\times \left(X_{s}-X_{\mathrm{ms}}\times \mathrm{TCF}\right)\;$$

(9) TCF={abs(R−ri), ifg≤gmaxCOFround(1+ri), ifg>gmaxCOF 

where $X_{j,i}^{\prime }$ is the updated solution; j = 1, 2,…, n; i = 1, 2,…, m. COF is the convection factor; R is the probability variable; R ∈ (0.6666, 1); r_i_ ∈ (0, 1) is a uniformly distributed random number; X_s_ is the temperature of the surrounding and X_ms_ is the mean temperature of the system; TCF is a temperature change factor.

#### 3.4.3. Radiation Phase

The solutions are updated in the radiation phase as per Equations (10) and (11) [48],

(10) Xj,i′={Xj,i+R×(Xk, i−Xj,i), iff(Xj)>f(Xk)Xj,i+R×(Xj,i−Xk, i), iff(Xj)<f(Xk) ;ifg≤gmaxRDF 

(11) Xj,i′={Xj,i+ri×(Xk, i−Xj,i), iff(Xj)>f(Xk)Xj,i+ri×(Xj,i−Xk, i), iff(Xj)<f(Xk) ;ifg>gmaxRDF 

where $X_{j,i}^{\prime }$ is the updated solution; j = 1, 2,…,n; i = 1, 2,…,m; j ≠ k; k ∈ (1, 2,…, n) and k is a randomly selected molecules; RDF is the radiation factor; R is the probability variable; R ∈ (0.3333, 0.6666); r_i_ ∈ (0, 1) is a uniformly distributed random number.

All the responses, such as MRR, SR, and the Kerf taper angle, were taken as positive integers during the execution of the HTS algorithm. Following machining, limits were used for AWJM process parameters during the execution of the algorithms.

Nozze Transverse Speed (T_v_): 150 mm/min ≤ T_v_ ≥ 250 mm/min.

Abrasive Flow Rate (A_f_): 300 g/min ≤ A_f_ ≥ 500 g/min.

Stand-off distance (S_d_): 1.5 mm ≤ S_d_ ≥ 3.5 mm.

For each of the objectives (MRR, SR, and the kerf taper angle), Table 7 displays the single-objective optimization using the HTS algorithm. If any objective reaches its ideal value, then other objectives are far from the desired levels. For example, for the maximization of MRR, corresponding values of SR and θ were not at their required levels. Similar observations can be made for other studied objective functions. i.e., for the minimization of SR and θ, the corresponding value of MRR was not at the required level of maximization. Table 7 also highlights the combination of the input variables is different for the individual objective functions. For the maximization of MRR, the input process parameters are T_v_ of 250 mm/min, A_f_ of 500 g/min, and S_d_ of 1.5 mm, while for the minimization of SR and θ, the input process parameters are T_v_ of 150 mm/min, A_f_ of 500 g/min, and S_d_ of 1.5 mm. This creates confusion for the manufacturer when finalizing the settings of the process parameters on the machine. Pareto optimal points are the solution for such complex problems. They provide a trade-off between output responses.

The simultaneous optimization of MRR, SR, and the kerf angle has been carried out by assigning equal weights of 0.33 to all the responses. Equation (12) shows the objective function for simultaneous optimization.

(12) $$\mathrm{Obj}\;=w_{1}\cdot \left(\mathrm{MRR}\right)+w_{2}\cdot \left(\mathrm{SR}\right)+w_{3}\cdot \left(\theta \right)$$

The simultaneous optimization result yielded response values of MRR, SR, and kerf angle of 0.2133 g/min, 3.50 µm, and 1.98, respectively at T_v_ of 192 mm/min, A_f_ of 500 g/min, and S_d_ of 1.5 mm. If all the response variables are of equal importance for the user, then this set of parameters of simultaneous optimization will be useful as they provide the optimal levels.

The multi-objective heat transfer search (MOHTS) algorithm is an extended version of the HTS algorithm, which can find the best optimal solution for the problems involving conflict between responses [49,50,51,52]. Initially, the MOHTS algorithm generates non-dominated solutions and stores it in an external archive. The e-dominance-based updating approach was used to check the solutions stored in the archive. The Pareto fronts are generated with the help of non-dominated solutions stored in the external archive. The grid-based approach with a fixed archive size is used in MOHTS for the archiving process. Every generation of the archive with the HTS algorithm was updated by the ε-dominance method. The space dimensions are presumed equal to the number of objective functions in the optimization problem. The boxes of size ε to ε are created in the space by slicing each dimension. The solutions that resulted during optimization are stored in these boxes. Further, the dominating boxes are kept and those that were dominated are removed. That is, the solution in those boxes were removed. Afterward, if the solution in the remaining box is more than one, then the dominated solution among them is removed. Finally, the non-dominated solution remains in the box and is retained in the archive.

**Figure 9:**
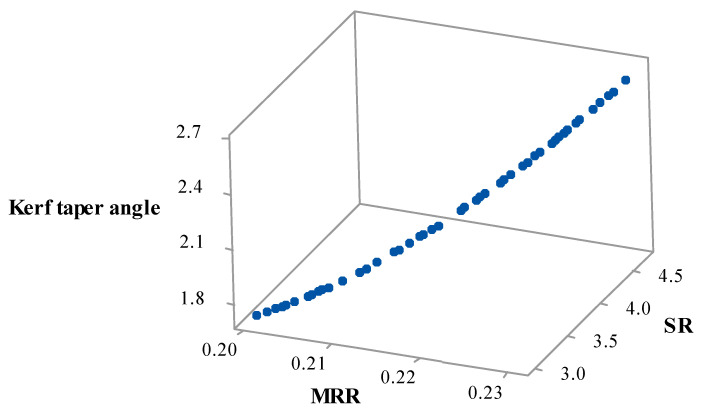
3D Pareto graph of MRR vs. SR vs. Kerf taper angle.

For the multi-objective optimization of the present study, the MOHTS algorithm was applied to find non-dominant Pareto optimal points. At the end of the 10,000-evolution function, the Pareto optimal points are obtained. Forty-eight Pareto points were generated, and each Pareto point provides a unique optimal solution. Table 8 shows the results of eight Pareto points along with their AWJM process parameters. The obtained Pareto optimal points were plotted in the 3D space as shown in Figure 9. In the 3D plot, the X-, Y-, and Z-axis represent MRR, SR, and the Kerf taper angle, respectively. Each Pareto point has its prediction for the studied responses and each prediction is a function of a combination of input parameter settings. The selection of a particular Pareto point by the operator depends on the specific requirement of the responses. The benefit of employing the MOHTS algorithm is that the Pareto points are non-dominated solutions and can be obtained in a single step. All the Pareto optimal points were obtained at the same values of A_f_ and S_d_. This suggests the A_f_ value of 500 g/min and S_d_ value of 1.5 mm provide optimal solutions. Table 9 shows the four randomly selected confirmatory trials of Pareto points along with their predicted values obtained from the MOHTS algorithm as well as experimentally measured response values of MRR, SR, and the kerf angle. The developed model and HTS algorithm can be considered capable as negligible variance was witnessed amongst the predicted and measured values.

**Figure 10:**
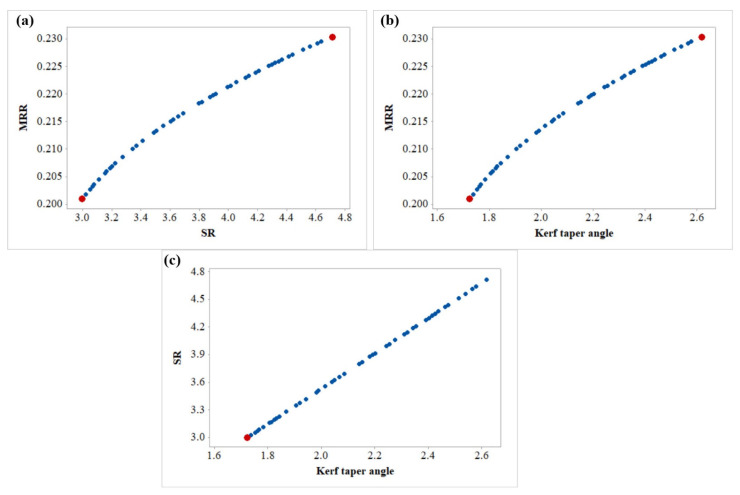
2D Pareto graph of (**a**) MRR vs. SR, (**b**) MRR vs. Kerf taper angle, and (**c**) SR vs. Kerf taper angle.

2D views of the Pareto points provide a better way to understand 3D Pareto points. The 2D views of MRR vs. SR, MRR vs. Kerf taper angle, and SR vs. Kerf taper angle are shown in Figure 10a–c, respectively. The point to note here is that these 2D views have an effect on the third variable. As can be observed from Figure 10a for the Pareto view of MRR vs. SR, the entire space is occupied by a discrete distribution of obtained Pareto points. Figure 10a highlights the maximum and minimum desired values of the MRR and SR as indicated by red points are 0.2304 g/min and 2.99 µm, respectively. The designer could face conflicting phenomena while dealing with MRR and SR. With a higher MRR, the SR would also be higher. Therefore, the designer must select the Pareto point that is a trade-off between these two values.

A similar situation can be seen in Figure 10b of the 2D Pareto graph of MRR vs. the Kerf taper angle. The maximum value of MRR and the minimum value of the Kerf taper angle were observed to be 0.2304 g/min and 1.72, respectively. For the maximum MRR, the kerf taper angle was also at the higher level and for the minimum kerf taper angle, MRR was also obtained at the lowest value. So, while selecting these two objectives, it depends on the user to select the Pareto points as per the requirement of response values.

Figure 10c shows the 2D Pareto graph of SR vs. the Kerf taper angle. As both responses are of the lower-the-better category, the Pareto point shown in red in Figure 10a will be the most useful for the industrial users as the lowest values of SR and the Kerf taper angle were obtained at the same parameters giving values of 2.99 µm and 1.72, respectively. Pursuant to this, the user selecting these two responses will obtain both the objectives at required levels at parametric settings at T_v_ of 150 mm/min, A_f_ of 500 g/min, and S_d_ of 1.5 mm.

### 3.5. Surface Morphology of Machined Components

**Figure 11:**
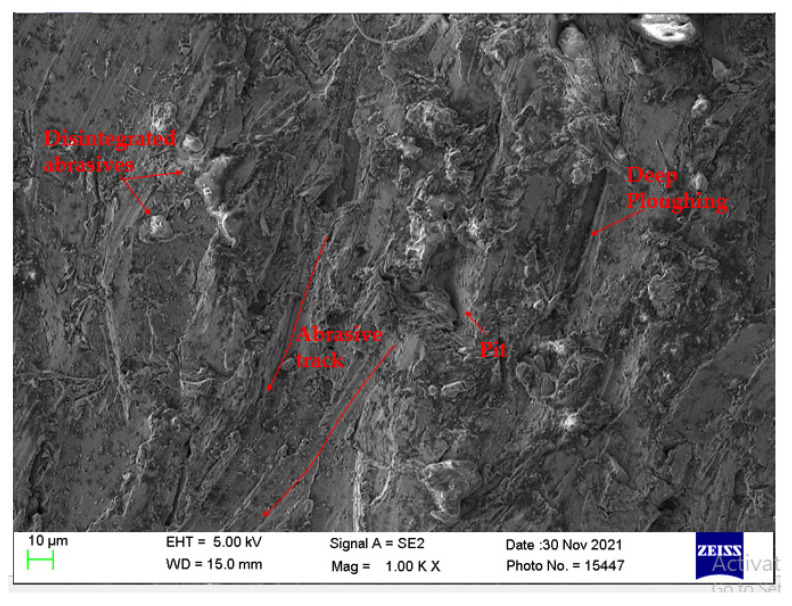
SEM micrograph of machined surface at T_v_ of 250 mm/min, A_f_ of 500 g/min, and S_d_ of 1.5 mm.

The surface morphology of machined surfaces observed under scanning electron microscopy is shown in Figure 11. The SEM images were taken at the top of the machined surface. The observed machined surfaces correspond to experiments performed with process parameters obtained from single-objective optimization. Figure 11 shows the machined surface obtained with process parameters T_v_ 250 mm/min, A_f_ 500 g/min, and S_d_ 1.5 mm. These parameters are obtained from the HTS algorithm when a single objective of maximizing MRR was considered. It can be elucidated from Figure 11 that the cut surface of the workpiece is strongly plowed. This is due to the shearing of the abrasive particles. For the maximization of MRR, T_v_ and A_f_ are at the highest level. This combination causes higher particle disintegration and embedding of fractured abrasive particles in the machined surface resulting in the higher impulse of abrasive particles [53]. As a result, a ploughing-like pattern (separate wear track) can be seen on the cut surface as shown in Figure 11. These patterns are longer and deeper indicating a rough surface [54]. Ploughing is one of the primary material-removal mechanisms in AWJM where the impinging high-velocity jet of water along with the abrasives scoop out the material along the flow direction of the jet. Similar observations are reported by Hascalik et al. [55] while studying Ti6Al4V alloy. They noticed that at a higher travel speed, the surface roughness also increases. This is due to less overlap of machining action and fewer abrasive particles to impinge the surface at a higher travel speed.

**Figure 12:**
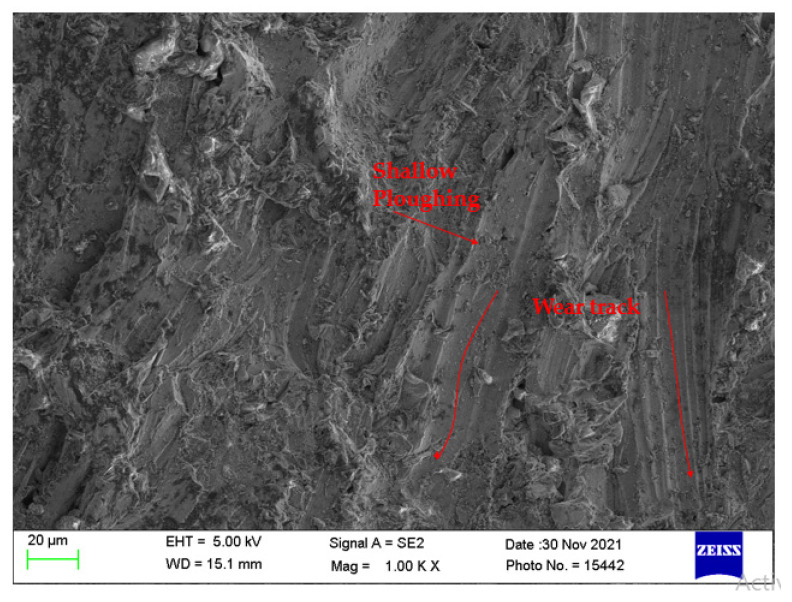
SEM micrograph of machined surface at T_v_ of 150 mm/min, A_f_ of 500 g/min, and S_d_ of 1.5 mm.

Figure 12 presents the machined surface obtained with process parameters T_v_ 150 mm/min, A_f_ 500 g/min, and S_d_ 1.5 mm. These parameters are obtained from the HTS algorithm when a single objective of minimizing SR was considered. These parameters produced roughness of 2.82 µm. The same process parameter setting was obtained from the HTS algorithm when a single objective of minimizing the kerf taper angle was considered. The kerf taper angle of 1.95 was obtained with the aforementioned parameter settings. Figure 12 reveals the reduced wear tracks on the machined surface. This may be due to lower particle disintegration at a lower nozzle travel speed, which produces sufficient kinetic energy during machining. This reduced abrasive contamination and produced a smooth surface.

**Figure 13:**
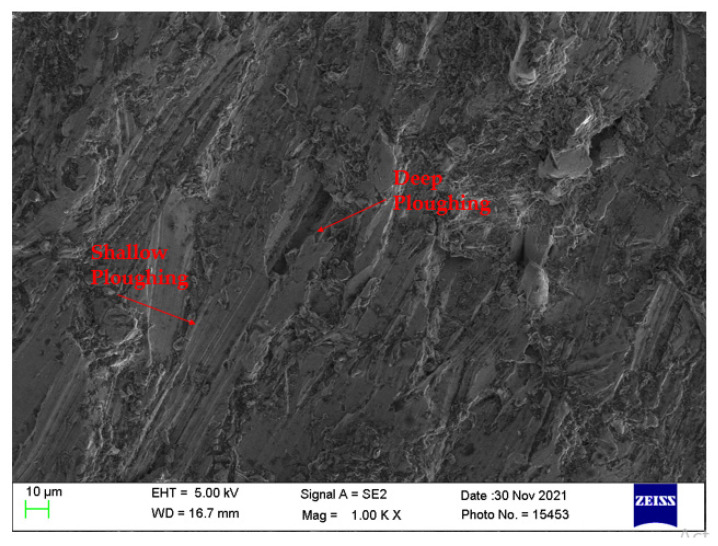
SEM micrograph of machined surface at T_v_ of 193 mm/min, A_f_ of 500 g/min, and S_d_ of 1.5 mm.

Figure 13 presents the machined surface obtained with process parameters T_v_ of 193 mm/min, A_f_ of 500 g/min, and S_d_ of 1.5 mm. These parameters are obtained from the HTS algorithm when simultaneous optimization was considered. It can be observed from the figure that the surface consists of shallow and deep ploughing marks. The above results coincide with Figure 6 whereby SR is directly correlated with the nozzle travel speed.

## 4. Conclusions

The present study investigated the effect of AWJM parameters (T_v_, A_f_, and S_d_) on responses of MRR, SR, and the Kerf taper angle for Ti6Al4V. Based on the work, the following important conclusions can be drawn:

- Mathematical regression models were generated using the RSM technique, and ANOVA results have shown the adequacy of the developed models.
- Normal probability, the significance of model terms, and the insignificance of lack-of-fit for all responses highlighted good prediction capabilities of the developed models of MRR, SR, and the kerf taper angle.
- Single-objective optimization results yielded a maximum MRR of 0.2304 g/min (at T_v_ of 250 mm/min, A_f_ of 500 g/min, and S_d_ of 1.5 mm), a minimum SR of 2.99 µm, and a minimum θ of 1.72 (both responses at T_v_ of 150 mm/min, A_f_ of 500 g/min, and S_d_ of 1.5 mm). Simultaneous optimization results, by considering an equal weightage of 0.33 to all responses, yielded MRR, SR, and θ values of 0.2133 g/min, 3.50 µm, and 1.98, respectively at T_v_ of 193 mm/min, A_f_ of 500 g/min, and S_d_ of 1.5 mm.
- 3D and 2D plots were plotted using Pareto optimal points, which highlighted the non-dominant feasible solutions. Every single Pareto point gives a unique solution and has a corresponding value of the input process parameter. Therefore, an operator can select a suitable point by just observing their required values of MRR, SR, and the kerf taper angle.
- The surface morphology revealed the material-removal mechanism in AWJM was due to ploughing, particle disintegration, and embedding of fractured abrasive particles in the machined surface.
- Different levels of input process parameters by varying the abrasives can be studied in the future to check the optimal levels of the AWJM responses.

## Figures and Tables

**Table 1:** Chemical composition (wt.%) of Ti6Al4V.

C	Fe	Al	N_2_	Cu	V	Ti
0.05	0.20	6.20	0.04	0.001	4.0	Balanced

**Table 2:** AWJM process parameters.

Process Parameter	Level (−1)	Level (0)	Level (1)
Nozze Transverse Speed (T_v_), mm/min	150	200	250
Abrasive Flow Rate (A_f_), g/min	300	400	500
Stand-off distance (S_d_), mm	1.5	2.5	3.5
Mesh size of abrasive	80		
Nozzle material	ROCTEC 100 Composite Carbide		
Nozzle diameter	1.02 mm		
Orifice material/diameter	Diamond/0.33 mm		
Impact angle of jet	90°		

**Table 3:** RSM BBD design along with experimentally measured values of responses.

Standard Order	Run Order	T_v_ (mm/min)	A_f_ (g/min)	S_d_ (mm)	MRR (g/min)	SR (µm)	θ (°)
8	1	250	400	3.5	0.1767	5.73	3.06
15	2	200	400	2.5	0.1706	4.32	2.31
4	3	250	500	2.5	0.2062	5.27	2.82
10	4	200	500	1.5	0.2250	3.58	2.06
12	5	200	500	3.5	0.2062	4.99	2.67
9	6	200	300	1.5	0.1076	4.76	2.53
11	7	200	300	3.5	0.1302	4.87	2.60
13	8	200	400	2.5	0.1650	4.26	2.28
6	9	250	400	1.5	0.1768	5.31	2.86
5	10	150	400	1.5	0.1375	3.59	2.007
1	11	150	300	2.5	0.1302	3.96	2.12
2	12	250	300	2.5	0.1303	5.68	3.04
3	13	150	500	2.5	0.2063	3.39	2.11
7	14	150	400	3.5	0.1768	4.24	2.27
14	15	200	400	2.5	0.1743	4.33	2.31

**Table 4:** ANOVA for MRR.

Source	DF	Adj SS	Adj MS	F Value	*p*-Value	Significance
Model	5	0.016149	0.003230	46.42	0.000	Significant
Linear	3	0.008332	0.002777	39.92	0.000	Significant
T_v_	1	0.000514	0.000514	7.39	0.024	Significant
A_f_	1	0.002825	0.002825	40.61	0.000	Significant
S_d_	1	0.000912	0.000912	13.11	0.006	Significant
2-way interaction	2	0.000814	0.000407	5.85	0.024	Significant
T_v_ × S_d_	1	0.000386	0.000386	5.55	0.043	Significant
A_f_ × S_d_	1	0.000429	0.000429	6.16	0.035	Significant
Error	9	0.000626	0.000070			
Lack of Fit	7	0.000582	0.000083	3.79	0.225	Insignificant
Pure Error	2	0.000044	0.000022			
Total	14	0.016775				
S = 0.008341, R-Sq. = 96.27%, R-Sq. (Adj.) = 94.19%						

**Table 5:** ANOVA for SR.

Source	DF	Adj SS	Adj MS	F Value	*p*-Value	Significance
Model	6	7.86108	1.31018	133.17	0.000	Significant
Linear	3	0.74036	0.24679	25.08	0.000	Significant
T_v_	1	0.04482	0.04482	4.56	0.065	Significant
A_f_	1	0.68120	0.68120	69.24	0.000	Significant
S_d_	1	0.34918	0.34918	35.49	0.000	Significant
Square	2	0.29148	0.14574	14.81	0.002	Significant
T_v_ × T_v_	1	0.17409	0.17409	17.69	0.003	Significant
S_d_ × S_d_	1	0.13805	0.13805	14.03	0.006	Significant
2-way interaction	1	0.42739	0.42739	43.44	0.000	Significant
A_f_ × S_d_	1	0.42739	0.42739	43.44	0.000	Significant
Error	8	0.07871	0.00984			
Lack of Fit	6	0.07599	0.01266	9.32	0.100	Insignificant
Pure Error	2	0.00272	0.00136			
Total	14	7.93978				
S = 0.0991, R-Sq. = 99.01%, R-Sq. (Adj.) = 98.27%						

**Table 6:** ANOVA for Kerf taper angle.

Source	DF	Adj SS	Adj MS	F Value	*p*-Value	Significance
Model	6	2.04354	0.34059	111.22	0.000	Significant
Linear	3	0.15244	0.05081	16.59	0.001	Significant
T_v_	1	0.01183	0.01183	3.86	0.085	Insignificant
A_f_	1	0.12179	0.12179	39.77	0.000	Significant
S_d_	1	0.10307	0.10307	33.66	0.000	Significant
Square	2	0.10476	0.05238	17.11	0.001	Significant
T_v_ × T_v_	1	0.04692	0.04692	15.32	0.004	Significant
S_d_ × S_d_	1	0.06521	0.06521	21.29	0.002	Significant
2-way interaction	1	0.07268	0.07268	23.73	0.001	Significant
A_f_ × S_d_	1	0.07268	0.07268	23.73	0.001	Significant
Error	8	0.02450	0.00306			
Lack of Fit	6	0.02372	0.00395	10.18	0.092	Insignificant
Pure Error	2	0.00078	0.00038			
Total	14	2.06804				
S = 0.0553, R-Sq. = 98.82%, R-Sq. (Adj.) = 97.93%						

**Table 7:** Single-objective optimization of responses.

Objective Function	T_v_ (mm/min)	A_f_ (g/min)	S_d_ (mm)	MRR (g/min)	SR (µm)	θ (°)
Maximum MRR	250	500	1.5	0.2304	4.71	2.61
Minimum SR	150	500	1.5	0.2009	2.99	1.72
Minimum θ	150	500	1.5	0.2009	2.99	1.72

**Table 8:** Pareto optimal points obtained from HTS algorithm.

Sr. No.	T_v_ (mm/min)	A_f_ (g/min)	S_d_ (mm)	MRR (g/min)	SR (µm)	θ (°)
1	250	500	1.5	0.2304	4.71	2.62
2	150	500	1.5	0.2009	3.00	1.72
3	219	500	1.5	0.2213	3.99	2.24
4	215	500	1.5	0.2201	3.91	2.20
5	247	500	1.5	0.2295	4.64	2.58
6	162	500	1.5	0.2044	3.11	1.78
7	232	500	1.5	0.2251	4.28	2.39
8	225	500	1.5	0.2230	4.12	2.31
9	222	500	1.5	0.2221	4.06	2.28
10	198	500	1.5	0.2151	3.60	2.04
11	195	500	1.5	0.2142	3.55	2.02
12	166	500	1.5	0.2056	3.15	1.81
13	228	500	1.5	0.2239	4.19	2.34
14	226	500	1.5	0.2233	4.14	2.32
15	156	500	1.5	0.2027	3.05	1.75
16	176	500	1.5	0.2086	3.28	1.87
17	172	500	1.5	0.2074	3.22	1.84
18	201	500	1.5	0.2159	3.65	2.07
19	229	500	1.5	0.2242	4.21	2.36
20	159	500	1.5	0.2036	3.08	1.77
21	153	500	1.5	0.2018	3.02	1.74
22	246	500	1.5	0.2292	4.61	2.57
23	181	500	1.5	0.2100	3.34	1.91
24	244	500	1.5	0.2286	4.56	2.54
25	242	500	1.5	0.2280	4.51	2.51
26	239	500	1.5	0.2272	4.44	2.48
27	238	500	1.5	0.2269	4.41	2.46
28	236	500	1.5	0.2263	4.37	2.44
29	235	500	1.5	0.2260	4.34	2.43
30	234	500	1.5	0.2257	4.32	2.41
31	233	500	1.5	0.2254	4.30	2.40
32	170	500	1.5	0.2068	3.20	1.83
33	169	500	1.5	0.2065	3.19	1.82
34	167	500	1.5	0.2059	3.17	1.81
35	220	500	1.5	0.2216	4.02	2.26
36	214	500	1.5	0.2198	3.89	2.19
37	213	500	1.5	0.2195	3.87	2.18
38	158	500	1.5	0.2033	3.07	1.76
39	210	500	1.5	0.2186	3.82	2.15
40	209	500	1.5	0.2183	3.80	2.14
41	203	500	1.5	0.2165	3.69	2.09
42	203	500	1.5	0.2165	3.69	2.09
43	186	500	1.5	0.2115	3.41	1.94
44	183	500	1.5	0.2106	3.37	1.92
45	199	500	1.5	0.2154	3.62	2.05
46	192	500	1.5	0.2133	3.51	1.99
47	191	500	1.5	0.2130	3.49	1.98
48	229	500	1.5	0.2242	4.21	2.36

**Table 9:** Confirmatory trials.

Sr. No.	T_v_ (mm/min)	A_f_ (g/min)	S_d_ (mm)	Predicted Values by HTS Algorithm			Experimentally Measured Values			% Deviation		
				MRR	SR	θ	MRR	SR	θ	MRR	SR	θ
1	250	500	1.5	0.2304	4.71	2.61	0.2395	4.57	2.52	3.79	3.06	3.57
2	150	500	1.5	0.2009	2.99	1.72	0.2101	2.83	1.78	4.37	5.65	3.37
10	192	500	1.5	0.2133	3.50	1.98	0.2194	3.69	2.06	2.78	5.14	3.88
38	158	500	1.5	0.2033	3.07	1.76	0.1997	3.15	1.84	1.80	2.53	4.34

## Data Availability

Data presented in this study are available in this article.
